# Supplementary material for: Genome-wide identification, characterization and gene expression of BES1 transcription factor family in grapevine (Vitis vinifera L.)
Source: Sci Rep. 2023 Jan 5;13:240. doi: 10.1038/s41598-022-24407-y (PMC9816167; doi:10.1038/s41598-022-24407-y)
Supplement: Supplementary file 3 — Supplementary Information. [file 41598_2022_24407_MOESM3_ESM.zip › Vvi_Atr/Vitis_vinifera.PN40024.v4.dna_sm.toplevel.fa.vs.Amborella_trichopoda.AMTR1.0.dna_sm.toplevel.fa.html/Atr-AmTr_v1.0_scaffold00165.html]

|  |  |  |  |  |  |  |  |  |  |  |  |  |  |
| --- | --- | --- | --- | --- | --- | --- | --- | --- | --- | --- | --- | --- | --- |
| Duplication depth | Reference chromosome | Collinear blocks | | | | | | | | | | | |
| 1 | Atr-ERN11982 |  | Vvi-Vitvi13g00492\_t001 |  |  |  |  |  |  |  |  |  |  |
| 1 | Atr-ERN11983 |  | \| \| |  |  |  |  |  |  |  |  |  |  |
| 3 | Atr-ERN11984 |  | \| \| |  | Vvi-Vitvi08g01293\_t001 |  | Vvi-Vitvi06g00563\_t001 |  |  |  |  |  |  |
| 4 | Atr-ERN11985 |  | Vvi-Vitvi13g04135\_t001 |  | \| \| |  | \| \| |  | Vvi-Vitvi13g02023\_t001 |  |  |  |  |
| 4 | Atr-ERN11986 |  | \| \| |  | \| \| |  | \| \| |  | \| \| |  |  |  |  |
| 4 | Atr-ERN11987 |  | \| \| |  | \| \| |  | \| \| |  | Vvi-Vitvi13g04126\_t001 |  |  |  |  |
| 4 | Atr-ERN11988 |  | \| \| |  | \| \| |  | \| \| |  | \| \| |  |  |  |  |
| 4 | Atr-ERN11989 |  | Vvi-Vitvi13g04133\_t001 |  | \| \| |  | Vvi-Vitvi06g00562\_t001 |  | \| \| |  |  |  |  |
| 4 | Atr-ERN11990 |  | \| \| |  | Vvi-Vitvi08g01292\_t001 |  | \| \| |  | Vvi-Vitvi13g04127\_t001 |  |  |  |  |
| 4 | Atr-ERN11991 |  | \| \| |  | \| \| |  | \| \| |  | \| \| |  |  |  |  |
| 4 | Atr-ERN11992 |  | \| \| |  | \| \| |  | \| \| |  | \| \| |  |  |  |  |
| 4 | Atr-ERN11993 |  | \| \| |  | \| \| |  | \| \| |  | \| \| |  |  |  |  |
| 4 | Atr-ERN11994 |  | Vvi-Vitvi13g04130\_t001 |  | \| \| |  | \| \| |  | \| \| |  |  |  |  |
| 4 | Atr-ERN11995 |  | Vvi-Vitvi13g00432\_t001 |  | \| \| |  | \| \| |  | \| \| |  |  |  |  |
| 4 | Atr-ERN11996 |  | \| \| |  | \| \| |  | \| \| |  | \| \| |  |  |  |  |
| 4 | Atr-ERN11997 |  | Vvi-Vitvi13g00431\_t001 |  | \| \| |  | \| \| |  | Vvi-Vitvi13g04129\_t001 |  |  |  |  |
| 4 | Atr-ERN11998 |  | Vvi-Vitvi13g00397\_t001 |  | \| \| |  | \| \| |  | \| \| |  |  |  |  |
| 4 | Atr-ERN11999 |  | \| \| |  | Vvi-Vitvi08g01291\_t001 |  | \| \| |  | \| \| |  |  |  |  |
| 4 | Atr-ERN12000 |  | \| \| |  | Vvi-Vitvi08g02230\_t001 |  | Vvi-Vitvi06g01729\_t001 |  | \| \| |  |  |  |  |
| 4 | Atr-ERN12001 |  | \| \| |  | \| \| |  | \| \| |  | \| \| |  |  |  |  |
| 4 | Atr-ERN12002 |  | \| \| |  | Vvi-Vitvi08g01288\_t002 |  | \| \| |  | \| \| |  |  |  |  |
| 4 | Atr-ERN12003 |  | Vvi-Vitvi13g02013\_t001 |  | Vvi-Vitvi08g01287\_t001 |  | Vvi-Vitvi06g00558\_t001 |  | \| \| |  |  |  |  |
| 4 | Atr-ERN12004 |  | Vvi-Vitvi13g00391\_t001 |  | \| \| |  | \| \| |  | \| \| |  |  |  |  |
| 4 | Atr-ERN12005 |  | \| \| |  | Vvi-Vitvi08g01285\_t001 |  | Vvi-Vitvi06g00556\_t001 |  | \| \| |  |  |  |  |
| 4 | Atr-ERN12006 |  | \| \| |  | \| \| |  | \| \| |  | \| \| |  |  |  |  |
| 4 | Atr-ERN12007 |  | \| \| |  | \| \| |  | \| \| |  | \| \| |  |  |  |  |
| 4 | Atr-ERN12008 |  | Vvi-Vitvi13g00390\_t001 |  | Vvi-Vitvi08g01282\_t001 |  | \| \| |  | \| \| |  |  |  |  |
| 4 | Atr-ERN12009 |  | \| \| |  | \| \| |  | \| \| |  | \| \| |  |  |  |  |
| 4 | Atr-ERN12010 |  | \| \| |  | \| \| |  | \| \| |  | \| \| |  |  |  |  |
| 4 | Atr-ERN12011 |  | Vvi-Vitvi13g00388\_t001 |  | Vvi-Vitvi08g01281\_t001 |  | \| \| |  | \| \| |  |  |  |  |
| 4 | Atr-ERN12012 |  | \| \| |  | \| \| |  | Vvi-Vitvi06g00555\_t001 |  | \| \| |  |  |  |  |
| 4 | Atr-ERN12013 |  | \| \| |  | \| \| |  | \| \| |  | \| \| |  |  |  |  |
| 4 | Atr-ERN12014 |  | \| \| |  | \| \| |  | \| \| |  | \| \| |  |  |  |  |
| 4 | Atr-ERN12015 |  | \| \| |  | \| \| |  | \| \| |  | \| \| |  |  |  |  |
| 4 | Atr-ERN12016 |  | \| \| |  | \| \| |  | \| \| |  | Vvi-Vitvi13g04135\_t001 |  |  |  |  |
| 4 | Atr-ERN12017 |  | \| \| |  | \| \| |  | \| \| |  | Vvi-Vitvi13g00490\_t001 |  |  |  |  |
| 3 | Atr-ERN12018 |  | \| \| |  | \| \| |  | \| \| |  |  |  |  |  |  |
| 3 | Atr-ERN12019 |  | \| \| |  | Vvi-Vitvi08g04251\_t001 |  | \| \| |  |  |  |  |  |  |
| 3 | Atr-ERN12020 |  | \| \| |  | \| \| |  | \| \| |  |  |  |  |  |  |
| 3 | Atr-ERN12021 |  | \| \| |  | \| \| |  | \| \| |  |  |  |  |  |  |
| 3 | Atr-ERN12022 |  | \| \| |  | \| \| |  | \| \| |  |  |  |  |  |  |
| 3 | Atr-ERN12023 |  | \| \| |  | \| \| |  | Vvi-Vitvi06g04180\_t001 |  |  |  |  |  |  |
| 3 | Atr-ERN12024 |  | \| \| |  | \| \| |  | \| \| |  |  |  |  |  |  |
| 3 | Atr-ERN12025 |  | \| \| |  | \| \| |  | \| \| |  |  |  |  |  |  |
| 3 | Atr-ERN12026 |  | Vvi-Vitvi13g00375\_t001 |  | \| \| |  | \| \| |  |  |  |  |  |  |
| 3 | Atr-ERN12027 |  | Vvi-Vitvi13g00365\_t001 |  | Vvi-Vitvi08g01273\_t001 |  | \| \| |  |  |  |  |  |  |
| 2 | Atr-ERN12028 |  | Vvi-Vitvi13g00364\_t001 |  |  |  | \| \| |  |  |  |  |  |  |
| 2 | Atr-ERN12029 |  | \| \| |  |  |  | \| \| |  |  |  |  |  |  |
| 2 | Atr-ERN12030 |  | \| \| |  |  |  | \| \| |  |  |  |  |  |  |
| 2 | Atr-ERN12031 |  | \| \| |  |  |  | \| \| |  |  |  |  |  |  |
| 2 | Atr-ERN12032 |  | Vvi-Vitvi13g00362\_t003 |  |  |  | Vvi-Vitvi06g00540\_t003 |  |  |  |  |  |  |
